# PPARγ Expression in Human Spermatozoa and Its Relationship with Seminal F_2_-Isoprostanes and Resolvin D1 in the Presence of Varicocele and Urogenital Infections

**DOI:** 10.3390/biology14020137

**Published:** 2025-01-28

**Authors:** Giulia Collodel, Elena Moretti, Caterina Marcucci, Laura Liguori, Daniela Marchini, Roberta Corsaro, Gabriele Centini, Cinzia Signorini

**Affiliations:** 1 Department of Molecular and Developmental Medicine, University of Siena, 53100 Siena, Italy; giulia.collodel@unisi.it (G.C.); caterin.marcucci@student.unisi.it (C.M.); laura.liguori@student.unisi.it (L.L.); r.corsaro@student.unisi.it (R.C.); gabriele.centini@unisi.it (G.C.); cinzia.signorini@unisi.it (C.S.); 2 Department of Life Sciences, University of Siena, 53100 Siena, Italy; daniela.marchini@unisi.it

**Keywords:** F_2_-isoprostanes, human spermatozoa, normozoospermia, PPARγ expression, resolvin D1, urogenital infections, varicocele

## Abstract

Peroxisome Proliferator-Activated Receptor Gamma (PPARγ) is a regulating agent in antioxidant response also involved in controlling inflammation. The impact of varicocele and urogenital infections on sperm PPARγ expression was studied. The PPARγ gene expression was investigated in spermatozoa of 26 normozoospermic men grouped according to their clinical conditions: normal semen parameters (N), normal semen parameters and varicocele (N + V), and normal semen parameters and urogenital infections (N + UI). Sperm PPARγ expression was correlated with F_2_-isoprostanes (F_2_-IsoPs), as markers of lipid peroxidation, and Resolvin D1 (RvD1), a pro-resolving mediator in inflammation. Sperm PPARγ expression was evaluated through comparative real-time PCR, and F_2_-IsoPs and RvD1 were quantified in the seminal plasma via GC/NCI-MS/MS and immunoassay, respectively. PPARγ expression correlates positively with sperm morphology and vitality and negatively with F_2_-IsoPs and RvD1. Sperm morphology positively correlates with vitality and negatively with F_2_-IsoP and RvD1 levels. Despite the normozoospermia in the three examined groups, sperm morphology and PPARγ expression were significantly reduced in N + V and N + UI groups compared to the N group. Additionally, F_2_-IsoP and RvD1 levels were elevated in N + V and N + UI patients. These data suggest that PPARγ expression is compromised by inflammation and lipoperoxidation, providing new insights to further explore new possibilities of targeted treatment of male infertility.

## Simple Summary

One of the most important knowledge acquisitions in the field of male infertility involves the relevant role of oxidative stress that, together with inflammation, is a common finding in many reproductive pathologies as varicocele, urogenital infections. Here, we studied the impact of these pathologies on sperm PPARγ, a transcription factor activated by polyunsaturated fatty acids and fatty acid derivatives and a regulating agent in antioxidant response. PPARγ causes a decrease in the production of proinflammatory cytokines, and it therefore has a significant involvement in controlling inflammation. Considering oxidative stress and inflammatory status, F_2_-IsoPs, prostaglandin-like products derived from arachidonic acid, and RvD1, a resolvin acting as lipid mediator of resolution of inflammation, were measured. The main results obtained in this study indicate a reduced PPARγ expression and high levels of F_2_-IsoPs and RvD1 in cases with varicocele and urogenital infections. Therefore, the reduced expression of PPARγ in human ejaculated spermatozoa is influenced by seminal oxidative stress and inflammation. PPARγ could be potentially modulated by its natural or artificial ligand, affording new therapeutic opportunities for a potential treatment for male infertility, increasing the performance of spermatozoa.

## 1. Introduction

Spermatogenesis is a complex process in which spermatogonia develop into spermatozoa within seminal tubules. This transformation relies on various cell types and an optimal fatty acid (FA) profile composing phospholipids, both in plasma membrane and seminal plasma [1,2].

Moreover, mammalian spermatozoa, during their maturation, undergo crucial metabolic modifications. Nuclear receptors found in spermatozoa may play a role in orchestrating these metabolic changes [3]; in particular, Peroxisome Proliferator-Activated Receptor Gamma (PPARγ) belongs to the nuclear hormone receptor superfamily, serving as a ligand-dependent transcription factor. PPARγ regulates the gene expression networks needed for cell proliferation, differentiation, morphogenesis, and metabolic balance.

Moreover, PPARγ serves as a critical transcriptional regulator modulating cellular glucose, lipid metabolism, and inflammatory responses [4].

Due to the dimensions of its binding cavity, the PPARγ ligand domain can interact with a range of natural or synthetic lipophilic acids, specifically FA or their derivatives, known as eicosanoids, including prostaglandins, leukotrienes, and other arachidonic acid (ARA) metabolites [5,6].

Aquila and colleagues [7] were the first group who reported the presence of PPARγ and the occurrence of PPARγ function in human-ejaculated spermatozoa. Indeed, it is also well known that PPARγ plays a critical role in ejaculated sperm motility and fertilization, since it is predominantly expressed in specific regions of these cells: in the apical region of the head, the post-acrosomal region, and mostly in the midpiece [8].

In addition, PPARγ’s non-nuclear localization in ejaculated sperm aligns with the hypothesis of the non-genomic mechanism of action in this specific cell type, and it might be the exclusive regulatory pathway in ejaculated spermatozoa [3,7,9].

Currently, semen analysis is the cornerstone of the evaluation of male fertilizing potential. According to the latest edition of WHO guidelines [10], the 5th percentile represents the lower reference value for normal semen parameters. In some cases, even in the presence of normal semen parameters, medical history, and physical examination, the men are still infertile. This occurs in approximately 15% of infertile men, who are classified as having unexplained infertility [11,12]. Varicocele and urogenital infections represent two of the most common pathologies that can hamper male fertility, and they share a common condition based on inflammation-mediated oxidative stress (OS) [13,14,15]. OS that plays a relevant role in male infertility arises from an imbalance between reactive oxygen species [16,17] and antioxidant defense, leading to sperm dysfunction and damage to proteins, lipids, and DNA [18,19].

F_2_-Isoprostanes (F_2_-IsoPs) are isomers of prostaglandin F_2_ alpha (PGF_2α_) belonging to the isoprostanoid class and resulting from the non-enzymatic oxidation of ARA, one of the molecular targets of OS damage. The mechanism leading to the formation of F_2_-IsoPs from the non-enzymatic oxidation of ARA and the nomenclature system to classify F_2_-IsoPs have been widely studied [20]. F_2_-IsoPs have emerged as a potent and reliable marker of in vivo lipid peroxidation (LPO, the term used to indicate the reaction of lipids with molecular oxygen and one of the most relevant processes in OS damage in biological systems), demonstrating their potential to accurately reflect OS status in various human diseases [20,21]. Among the 64 compounds that are generated by the mechanism for the formation of F_2_-IsoPs [20], 8-epi-PGF_2α_ is one of the most represented and is one of the most evaluated isomers for the measurement of F_2_-IsoPs.

In the management of inflammatory processes, specialized pro-resolving lipid mediators facilitate the resolution of inflammation. Specialized pro-resolving lipid mediators are formed by the enzymatic oxygenation of polyunsaturated fatty acids (PUFA) and, in addition to lipoxins, protectins, and maresins, include resolvins. As a component of specialized pro-resolving lipid mediators, Resolvin D1 (RvD1), derived from docosahexaenoic acid, exerts anti-inflammatory and pro-resolving activities in acute inflammation [22]. Signorini et al. [23] suggested a link between RvD1, OS, inflammation, and FA profile in human spermatozoa. That study provided strong evidence that RvD1 can represent a potential biomarker for identifying pathological seminal conditions.

The aim of this study was to quantify the PPARγ relative mRNA levels in spermatozoa from patients with normal semen parameters (above the 5th percentile according to the WHO [10]), and with normal semen parameters and varicocele or positive semen culture (classified as urogenital infections). This approach allows us to explore whether the presence of these reproductive pathologies can influence PPARγ expression. In the seminal plasma of the same cases, F_2_-IsoPs and RvD1 were dosed in order to investigate the relationship between these two markers of OS and inflammation, respectively, and PPARγ expression.

## 2. Materials and Methods

### 2.1. Patients

The semen samples were obtained from 26 selected donors (aged between 28 and 39 years) recruited at the Department of Molecular and Developmental Medicine, University of Siena.

Samples with semen parameters above the 5th percentile of the WHO guidelines for semen analysis [10] were included in this research. The participants had a normal karyotype, showed a BMI < 25 kg/m^2^, and had normal serum hormonal levels (follicle-stimulating hormone, luteinizing hormone, testosterone, prolactin); they did not have any chronic diseases and were not receiving radiotherapy, chemotherapy, or medication. Donors did not take oral antioxidant supplements for at least 6 months before the study, or take recreational drugs or consume alcohol. All subjects with a smoking habit were excluded.

Among these 26 normozoospermic donors, 7 individuals had urogenital infections and 9 had varicocele. The specific infectious agent and, for patients with varicocele, the grade of the pathology, were recorded in each patient’s medical history. The donors were grouped according to their clinical conditions:

Normal parameters with negative medical history (no problems declared during anamnesis) and normal clinical investigation in terms of absence of varicocele and urogenital infections (n° 10, N);

Normal parameters and urogenital infections (n° 7, N + UI);

Normal parameters and varicocele (n° 9, N + V).

The participants were informed about the study protocol and their privacy was ensured. They signed an informed consent form before participating in this research, agreeing that their semen samples could be used for scientific purposes. The study was conducted in accordance with the Declaration of Helsinki, and the protocol was approved by the Ethic Committee Siena University Hospital (ID CEAVSE 25612).

### 2.2. Semen Analysis and Sample Preparation

The semen samples were collected in sterile containers by masturbation after 3–5 days of sexual abstinence, and the semen was preserved from temperature fluctuations and it was ensured that it was delivered to the laboratory within 30 min of collection.

Semen analysis was performed following the WHO guidelines [10]. Normal semen parameters typically fall above the 5th percentile.

After semen analysis, the samples were centrifuged at 400× *g* for 10 min and spermatozoa and the seminal plasma were stored separately at −80 °C.

### 2.3. Peroxisome Proliferator-Activated Receptor Gamma (PPARγ) Determination

The determination of PPARγ expression was performed using PPARγ relative mRNA levels, starting with mRNA extraction from frozen sperm cell pellets using the RNeasy^®^ Plus Universal Mini Kit (QIAGEN, Hilden, Germany), following the protocol provided by the manufacturer. Briefly, each sample was lysed by adding QIAzol Lysis Reagent, and gDNA Eliminator Solution was also added. After centrifugation at 12,000× *g* for 15 min at 4 °C, RNA was further purified using a RNeasy Mini spin column (QIAGEN, Hilden, Germany). Following RNA extraction, quantification was performed using NanoDrop™ One (Thermo Scientific™ NanoDrop™ One Microvolume UV-Vis Spectrophotometers, Fisher Scientific, Waltham, MA, USA. To synthesize single-stranded cDNA from the extracted RNA, reverse transcription (RT) was performed using a High-Capacity cDNA Reverse Transcription Kit (Applied Biosystems, Waltham, MA, USA). The reaction volume was set to 20 µL, without the use of an RNase Inhibitor. A 2× RT Master Mix was prepared as indicated in the kit instructions. For each sample, 500 ng of RNA was reverse-transcribed in a final reaction volume to 20 µL. The reaction mix was centrifuged briefly to remove air bubbles and kept on ice before loading into the thermal cycler (PTC-200 Thermal Cycler, MJ Research, Waltham, MA, USA), which was programmed according to the instructions.

Comparative real-time PCR was performed using the QuantStudio™ 5 Real-Time PCR System (Thermofisher, Waltham, MA, USA). The analysis was carried out in a 96-well plate; each sample was amplified in triplicate for enhanced accuracy for both PPARγ and glyceraldehyde-3-Phosphate Dehydrogenase (GAPDH), the housekeeping gene used as a control. The corresponding primers were purchased from Bio-Rad (Assay ID qHsaCED0044425—PPARgamma; Assay ID qHsaCED0038674—GAPDH, Biorad, Hercules, CA, USA).

Reagents are prepared following the SYBR^®^ Green Assay protocol (Bio-Rad, Hercules, CA, USA). The number of cycles and temperatures and the time for each cycle were set according to the manufacturer’s instruction. In this study, PPARγ expression results were normalized to the GAPDH mRNA level. The relative quantification of the comparative real-time PCR data was determined using the double delta C_T_ (2^−ΔΔC^_T_) method [24].

### 2.4. F_2_-Isoprostane (F_2_-IsoP) Determination

After thawing, the levels of total F_2_-IsoPs in seminal plasma were quantified using a procedure also applied in other types of biological samples [25]. Briefly, basic hydrolysis was carried out in the presence of 1N KOH by means of incubation at 45 °C for 45 min. Then, 1N HCl was added to acidify and 500 pg of PGF_2α_-d_4_ was also added to each sample. Afterward, each sample was purified by two solid phase extractions, the first using an octadecylsilane (C_18_) cartridge, and the second using an aminopropyl (NH_2_) cartridge. In the subsequent derivatization process, the F_2_-IsoP carboxylic group was derivatized into pentafluorobenzyl esters, and the hydroxyl groups were converted to trimethylsilyl ethers.

The final quantification of F_2_-IsoPs was performed using gas chromatography/negative ion chemical ionization tandem mass spectrometry (GC/NICI-MS/MS) analysis (Trace GC and PolarisQ Ion Trap, Thermo Finnigan, San Jose, CA, USA). F_2_-IsoPs were quantified by measuring the *m*/*z* 299 ion, referred to as the 8-iso-PGF_2α_, which is known as a relevant F_2_-IsoPs isomer. Results are reported as ng/mL.

### 2.5. Resolvin D1 (RvD1) Determination

In seminal plasma, the levels of RvD1 were quantified using a double-antibody sandwich, Enzyme-Linked Immunosorbent Assay (MyBioSource, San Diego, CA, USA). The assay utilized a pre-coated anti-RvD1 monoclonal antibody as the capture antibody and a biotin-labeled polyclonal antibody as the detecting antibody. The optical density (OD) was measured at 450 nm.

### 2.6. Statistical Analysis

Statistical analysis was performed with SPSS version 17.0 for Windows software package (SPSS Inc, Chicago, IL, USA). Data were reported as medians and interquartile range (IQR 25°–75° percentiles). Spearman’s rho (r) coefficient was calculated to measure the correlation between variables for all enrolled cases. The comparisons between groups (N, N + V, N + UI) were evaluated using a Kruskal–Wallis test, followed, for significant cases only, by Dunnet’s post hoc test applied for pairwise comparisons. A *p* value < 0.05 (two-tailed) was considered statistically significant.

## 3. Results

Semen volume, sperm concentration, progressive motility, normal morphology, and vitality were assessed in semen samples of 26 individuals with normal semen parameters (>5th percentile, [10]). F_2_-IsoPs and RvD1 were dosed in seminal plasma, and PPAR*γ* expression was assessed in spermatozoa.

In Table 1, the medians (25° and 75° percentiles) for each variable are shown.

To understand the possible correlations among the studied variables, we used Spearman’s rank correlation coefficient, considering the whole population of interest. In Table 2, the significant correlations are shown.

Regarding sperm characteristics, sperm morphology positively correlated with sperm vitality (r = 0.54, *p* < 0.01) and negatively with F_2_-IsoP levels (r = −0.40, *p* < 0.05) and RvD1 (r = −0.46, *p* < 0.05). RvD1 also showed a negative correlation with sperm vitality (r = −0.48, *p* < 0.05).

PPARγ expression had a positive correlation with sperm morphology and vitality (respectively, r = 0.45, *p* < 0.05; r = 0.58, *p* < 0.01) and it was negatively correlated with F_2_-IsoP levels (r = −0.54, *p* < 0.01) and RvD1 (r = −0.60, *p* < 0.01).

The same variables were compared in the three groups (Table 3): the group with normal semen parameters (N), the group with normal parameters and urogenital infections (N + UI), and the group with normal parameters and varicocele (N + V).

Sperm morphology was significantly reduced in the cases of N + V and N + UI with respect to the N group (*p* < 0.05); sperm vitality was also significantly decreased in N + UI with respect to the N group (*p* < 0.001). The levels of F_2_-IsoPs were significantly lower in the N group with respect to those measured in the N + UI group (*p* < 0.05) and N + V (*p* < 0.001). The N + V group showed significantly higher levels of F_2_-IsoPs than the N + UI group (*p* < 0.05).

Despite the condition of normozoospermia, the N + UI patients (*p* < 0.01) and N + V group (*p* < 0.01) showed higher RvD1 levels with respect to those measured in group N.

**Figure 1:**
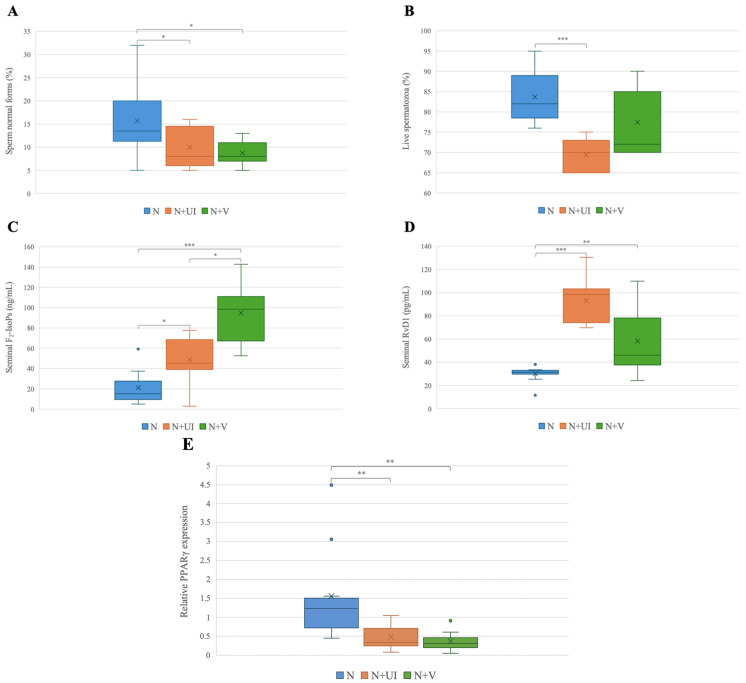
Box plots representing the sperm morphology % (**A**), the sperm vitality % (**B**), the seminal F_2_-IsoPs pg/mL(**C**), the seminal RvD1 pg/mL (**D**), and sperm PPARγ relative expression (**E**) in the three analyzed groups. N = donors with normal parameters with negative anamnesis and clinical investigation; N + UI = donors with normal parameters and urogenital infections; N + V = donors with normal parameters and varicocele. * *p* < 0.05, ** *p* < 0.01, *** *p* < 0.001.

The PPARγ expression was significantly increased in the N + UI and N + V groups compared to that detected in the N group (*p* < 0.01) (Figure 1).

## 4. Discussion

In this study, the expression of PPARγ was evaluated in the spermatozoa of men with normal semen parameters (N), and in men with normal semen parameters affected by pathological reproductive conditions such as varicocele (N + V) or urogenital infections (N + UI). The use of samples with normal sperm parameters allows the study of the direct impact of the pathology in PPARγ expression, OS, and inflammatory status.

PPARγ belongs to the family of nuclear receptors; however, while other nuclear receptors have a single specific ligand, numerous natural PPARγ ligands, in particular FAs or their derivatives eicosanoids, are available. Due to the close relationship between PPARγ and lipid metabolites, in this study, we considered the seminal levels of F_2_-IsoPs, oxygenated products from ARA metabolism, and RvD1, a resolvin acting as a lipid mediator of resolution of inflammation. Cumulative findings indicate that seminal F_2_-IsoPs and RvD1 are promising biomarkers for the evaluation of semen quality. Fertile men have lower levels of seminal F_2_-IsoPs and RvD1 with respect to those observed in infertile men with reproductive pathologies associated with inflammation [23,26]. Polvani’s group [27] proposed that PPARγ exerts an anti-inflammatory effect mediated by a non-genomic pathway. It is known that by stimulating PPARγ through its ligands, a decrease in the production of proinflammatory cytokines is achieved and this reduction is facilitated mainly through the suppression of NF-kB [28].

PPARγ is expressed in human ejaculated spermatozoa [7], in the Sertoli cells and spermatocytes, indicating a critical role of this receptor in spermatogenesis [8].

The present study indicated that the expression of PPARγ in spermatozoa was associated with seminal lipid markers of inflammation and OS. Sperm PPARγ showed negative correlations with seminal RvD1 and F_2_-IsoPs, suggesting that this transcription factor is reduced in conditions of inflammation and LPO in human semen.

Remarkably, PPARs are a group of fatty acid-regulated transcription factors that control both lipid metabolism and inflammation, and for PPARγ, unsaturated fatty acids are listed as endogenous ligands. In particular, the main mechanisms responsible for the anti-inflammatory activity of PPARγ is the crosstalk with the nuclear factor erythroid 2-related factor 2 (NRF2) [29]. In addition, several antioxidant genes are under transcriptional control of PPARs [30] and the cooperation between the transcription factors, Nrf2 and PPARγ, against oxidative damage has been described [31]. Interestingly, PPARγ has been reported to be involved in the regulation of anti-inflammatory mechanisms and OS in the brain [32]. Thus, PPARγ is indicated as an anti-inflammatory and antioxidant gene [27].

The positive relationship between sperm PPARγ and sperm morphology and vitality, observed in the present study, supports its protective action on human spermatozoa. The link between PPARγ and vitality was also observed in pig spermatozoa [33]. We did not find any relationship between progressive motility and PPARγ expression, probably due to the reduced number of cases included in the study. Conversely, other authors reported that sperm PPARγ mRNA expression and protein levels showed a tendency to be higher in normozoospermic than asthenozoospermic men [3], indicating a positive link between PPARγ and human sperm motility.

The comparison between the three considered categories (N; N + V; N + UI) showed a reduced PPARγ expression in N + V and N + UI groups with respect to that detected in the N group, suggesting that reproductive pathologies, such as varicocele or urogenital infections, in cases where increased F_2_-IsoP and RvD1 levels indicate the presence OS and inflammation, can influence PPARγ expression. The increased RvD1 values indicate that the presence of OS and inflammation push for the resolution of this process in the two pathological conditions investigated.

Despite normal sperm parameters, the percentages of sperm with normal morphology and normal vitality were reduced in the presence of varicocele and urogenital infections, suggesting a protective role of PPARγ in the regulation of sperm maturation and morphology. To support this hypothesis, studies have shown a significant decrease in PPARα expression in the testes of a mice model of varicocele [34]. It is also reasonable to suppose that reduced PPARγ activity might contribute to abnormal sperm morphology, since PPARγ shares a similar modular structure with steroid receptors that are under-expressed in men affected by varicocele [35].

The morphology of spermatozoa is a parameter that indicates their overall health and function, and the presence of morphological abnormalities results in an impairment of sperm’s ability to move, survive, and fertilize the oocyte. Defective morphology can make them more vulnerable to damage from external factors such as changes in pH, temperature, and the presence of ROS [36,37,38].

The sperm concentration was not significantly different in the three groups. However, the N + UI and N + V groups, despite the presence of pathologies, showed a sperm concentration higher than that observed in the N group, although non-significantly. In the analyzed cases, all the other parameters except for sperm concentration were decreased in the presence of pathologies, meaning that in the case of normozoospermia, the sperm concentration is the sperm parameter less affected by these pathologies, at least in the selected cases.

Our results regarding the role of PPARγ in regulating male reproduction are also validated by a study that reported a significant downregulation of PPARγ expression in both the testes and kidneys of hypothyroid rats. It is known that untreated hypothyroidism may result in several health problems, including infertility and, in this case, PPARγ inhibition contributed to testicular damage, exacerbating OS and inflammation [39].

PPARγ down-regulation concomitant with high levels of OS is well documented both in vitro and in vivo [40,41,42], while PPARγ activation inhibits the inflammatory responses up-regulating antioxidant genes such as catalase and glutathione peroxidase [43].

The experimental data suggest that during OS and inflammation, ligands of PPARγ, such as oxidized lipids and 15-deoxy-delta-prostaglandin J_2_ (15d-PGJ_2_), are produced. This implies that PPARγ, if already expressed, could serve as one of the initial responders to OS by directly triggering the production of antioxidant compounds, inhibiting prooxidants while simultaneously safeguarding cells against apoptosis [27]. In the spermatozoon, the central role of PPARγ in limiting OS and consequently ROS damage might mediate rapid responses to OS and adjustments to such environments to preserve sperm function.

The level of PPARγ expression found in ejaculated sperm might be influenced by altered spermatogenesis. In fact, spermatozoa are highly differentiated cells, with a low transcriptional activity, containing a wide variety of both coding and non-coding RNA molecules packaged during spermatogenesis and epididymal transit [44]. However, several evidence coming from the literature cannot exclude that mature spermatozoa can accomplish some level of transcription [45,46]. It is reported that the transcripts can vary during sperm freezing [47,48], suggesting that also the modifications of the environment, for example, seminal plasma components, can have an impact on gene expression.

At this purpose, both varicocele and urogenital infections likely create an inflammatory environment within the male reproductive tract, which can increase ROS production suppressing PPARγ activity as discussed above. PPARγ is a regulator of lipid metabolism and may be positively influenced by n-3 PUFA dietary sources supporting sperm maturation and reducing F_2_-IsoP levels [49].

This is the first study reporting the relationship between RvD1 and PPARγ in human semen and spermatozoa. In other systems as lung or human placenta, the ability of RvD1 in attenuating inflammatory process by a mechanism that is partially dependent on PPARγ activation was already observed [50,51]. The influence of diet on the concentration of RvD1 was recently demonstrated in a rabbit model [52].

In conclusion, reduced expression of PPARγ in human ejaculated spermatozoa appears to be influenced by the presence of seminal OS, this could play a role in reproductive pathological conditions associated with OS as varicocele, urogenital infections, or others.

Nevertheless, it is important to highlight that PPARs are regulated by multiple molecules that can act as co-activators or co-repressors [53]. Thus, further molecules and factors might be involved in the relationship between of PPARγ and LPO in male infertility.

We are aware that the study involves a small number of patients, which will need to be increased, however the strict selection criteria make the groups reliable.

Future perspectives can include the evaluation of experimental or clinical conditions, for example idiopathic infertility, that lead to investigating whether the variation in PPARγ expression and the increase in LPO and RvD1 are concurrent events, or which is dependent and secondary to the other.

PPARγ protein is highly expressed in ejaculated spermatozoa and the possibility to use natural or synthetic ligands is a relevant strategy to improve the sperm performance [44]. Given the close link between energy balance and reproduction, activation of PPARγ may have promising metabolic implications in male reproductive functions.

## Figures and Tables

**Table 1:** Considered parameters in the studied population. Median (25° and 75° percentiles) of the considered variables in the 26 cases included in this study.

Variables	Median (25°–75° percentiles)
Semen volume (mL)	3.50 (2.70–4.50)
Sperm concentration (10^6^ per mL)	75.00 (44.00–17.80)
Sperm progressive motility (PR, %)	47.50 (42.00–56.60)
Normal sperm morphology (%)	11.00 (6.75–14.25)
Sperm vitality (%)	77.00 (70.00–85.25)
Seminal F_2_-Isoprostanes (F_2_-IsoPs, ng/mL)	48.87 (16.05–82.01)
Seminal Resolvin-D1 (RvD1, pg/mL)	40.65 (31.63–83.32)
PPARγ (relative mRNA expression)	0.61 (0.29–1.10)

**Table 2:** Significant correlations are reported, * *p* < 0.05, ** *p* < 0.01.

	Normal Sperm Morphology (%)	Sperm Vitality (%)	Seminal F_2_-Isoprostanes (F_2_-IsoPs, ng/mL)	Seminal Resolvin-D1 (RvD1, pg/mL)
Sperm vitality (%)	r = 0.54 ** 95% C. I. = 0.19 to 0.78			
Seminal F_2_-Isoprostanes (F_2_-IsoPs, ng/mL)	r = −0.40 * 95% C. I. = −0.69 to −0.01			
Seminal Resolvin-D1 (RvD1, pg/mL)	r = −0.46 * 95% C. I. = −0.73 to −0.08	r = −0.48 * 95% C. I. = −0.74 to −0.11		
PPARγ (relative mRNA expression)	r = 0.45 * 95% C. I. = 0.06 to 0.72	r = 0.58 ** 95% C. I. = 0.24 to 0.80	r = −0.54 ** 95% C. I. = −0.77 to −0.18	r = −0.60 ** 95% C. I. = −0.81 to −0.27

**Table 3:** Medians (interquartile range) of sperm characteristics, seminal F_2_-isoprostanes (F_2_-IsoPs), seminal Resolvin-D1 (RvD1), and sperm PPARγ expression evaluated in semen samples of 26 men, divided into three groups according to their clinical condition (normal parameters: 10 men, N; normal parameters and urogenital infections: 7 men (N + UI); normal parameters and varicocele: 9 men, N + V). Statistics are also reported: NS non-significant, * *p* < 0.05, ** *p* < 0.01, *** *p* < 0.001.

Variables	N	N + UI	N + V	Kruskal–Wallis Test	Dunnet’s Post Hoc Test
Semen volume (mL)	3.70 (2.60–4.75)	3.50 (3.25–5.75)	2.80 (2.40–3.80)	NS	/
Sperm concentration (10^6^ per mL)	54.50 (44.25–112.25)	86.00 (54.00–135.00)	82.00 (43.00–110.00)	NS	/
Sperm progressive motility (PR, %)	50.00 (47.25–61.00)	42.00 (40.00–54.50)	46.00 (44.00–48.00)	NS	/
Normal sperm morphology (%)	13.50 (11.25–20.00)	8.00 (6.00–14.50)	8.00 (7.00–11.00)	*p* < 0.05	N + V vs. N * N + UI vs. N *
Sperm vitality (%)	82.00 (78.50–89.00)	70.00 (65.00–73.00)	72.00 (70.00–85.00)	*p* < 0.001	N + UI vs. N ***
Seminal F_2_-IsoPs (ng/mL)	15.14 (9.42–27.87)	45.27 (38.94–68.49)	98.49 (67.11–111.05)	*p* < 0.01	N + UI vs. N * N + V vs. N *** N + V vs. N + UI *
Seminal RvD1 (pg/mL)	31.38 (29.85–33.01)	98.49 (74.07–103.25)	45.96 (37.65–78.26)	*p* < 0.001	N + UI vs. N *** N + V vs. N **
PPARγ (relative mRNA expression)	1.24 (0.72–1.51)	0.34 (0.25–0.71)	0.31 (0.20–0.46)	*p* < 0.01	N + UI vs. N ** N + V vs. N **

## Data Availability

The data generated and analyzed during this study are included in this published article and are available from the corresponding author.
